# Denture Stomatitis and Candida albicans in the Indian Population: A Systematic Review and Meta-Analysis

**DOI:** 10.7759/cureus.45182

**Published:** 2023-09-13

**Authors:** Harkanwal Preet Singh, Pardeep Bansal, Thippeswamy SH

**Affiliations:** 1 Department of Oral Pathology and Microbiology, Dasmesh Institute of Research and Dental Sciences (Baba Farid University of Health Sciences), Faridkot, IND; 2 Department of Prosthodontics, Dasmesh Institute of Research and Dental Sciences (Baba Farid University of Health Sciences), Faridkot, IND

**Keywords:** meta-analysis, systematic review, indian population, risk factors, prevalence, candida albicans, denture stomatitis

## Abstract

Denture stomatitis (DS), a common oral condition among denture wearers, is frequently associated with *Candida albicans *(*C. albicans*) colonization. This systematic review and meta-analysis aimed to provide a comprehensive assessment of DS prevalence and its relationship with *C. albicans* in the Indian population. We conducted a thorough search of multiple databases for studies without any limitation to the publication timeframe, using the Preferred Reporting Items for Systematic Reviews and Meta-Analyses (PRISMA) guidelines. Eligible studies were assessed for quality and included in the meta-analysis. Data regarding DS prevalence, risk factors, and *C. albicans* colonization were extracted and analyzed. A total of four studies comprising 415 participants were included in the review. The overall odds ratio (OR) and risk ratio (RR) for the prevalence of *C. albicans* in DS patients were 0.75 (95% CI 0.56 to 0.99) and 0.83 (95% CI 0.70 to 1.00), respectively, indicating a statistically significant association between DS and *C. albicans* colonization. Several risk factors, including continuous denture wearing, advanced denture age, poor denture hygiene, and high sugar intake, were identified as contributing to DS development. This systematic review and meta-analysis highlight the substantial burden of DS and its association with *C. albicans* colonization in the Indian population. The findings emphasize the need for comprehensive oral care, improved denture hygiene, dietary counseling, and interventions to enhance salivary flow in denture wearers to mitigate the risk of DS. These insights can inform healthcare providers and policymakers to develop targeted strategies for DS prevention and management in India. Furthermore, this study emphasizes the significance of oral health awareness and preventive strategies in groups with a high frequency of denture usage in a larger population.

## Introduction and background

Denture stomatitis (DS) stands as a pervasive inflammatory condition of the oral mucosa, marked by its multifactorial etiological origins and its frequent manifestation in individuals reliant on dentures [1]. Characterized by an array of erythematous lesions, at times accompanied by discomfort, DS primarily affects the mucosal tissues beneath denture prostheses. *Candida albicans *(*C. albicans*), a commensal fungal inhabitant within the oral milieu, emerges as a pivotal etiological factor within this pathological framework [2]. The inception of this affliction unfolds as an outcome of the adherence and subsequent prolific propagation of microbial communities, exemplified by *C. albicans*, upon the acrylic substrates constituting dentures. The sequelae of such microbial colonization often precipitate painful inflammatory responses within the contiguous oral mucosal terrains [3-4]. Dentures, notably encompassing acrylic-based complete or partial prostheses, find pervasive utilization in the realm of dental care, predominantly targeting edentulous cohorts, with a notable prevalence among the elderly demographic [5].

One of the primary roles of dentures in DS is their potential to cause irritation and inflammation of the oral tissues [6]. Ill-fitting dentures can exert pressure on the soft tissues of the mouth, leading to friction and sore spots [7]. This continuous irritation creates an environment where fungal infections, notably *C. albicans*, can thrive, contributing to the development of DS [8-11]. Conversely, properly fitting and well-maintained dentures can help prevent denture stomatitis. Dentures that fit well distribute pressure evenly across the oral tissues, reducing the risk of tissue damage and irritation [12]. Regular cleaning and proper hygiene practices for dentures are also essential, as they prevent the buildup of bacteria and yeast on the denture surface, which is a common contributing factor to DS [13].

DS is a condition of substantial clinical importance as it not only affects the quality of life of denture wearers but also raises concerns about its potential link to systemic health issues [2]. Understanding the dynamics between DS and *C. albicans* is of paramount importance not only for oral healthcare providers but also for public health practitioners and policymakers [14]. Despite the known association between DS and *C. albicans*, the prevalence and specific characteristics of this association within the Indian population remain to be elucidated.

The objectives of this systematic review were twofold: first, to synthesize the existing body of literature encompassing studies conducted within the Indian population that have investigated the prevalence of *C. albicans* among DS patients, and second, to quantitatively assess this association. By rigorously reviewing and analyzing the available data, this study seeks to provide a comprehensive overview of the prevalence of *C. albicans* in DS patients in India, shedding light on the potential implications for oral healthcare practices, patient management, and future research endeavors.

## Review

Materials and methods

Review Design

**Figure 1:**
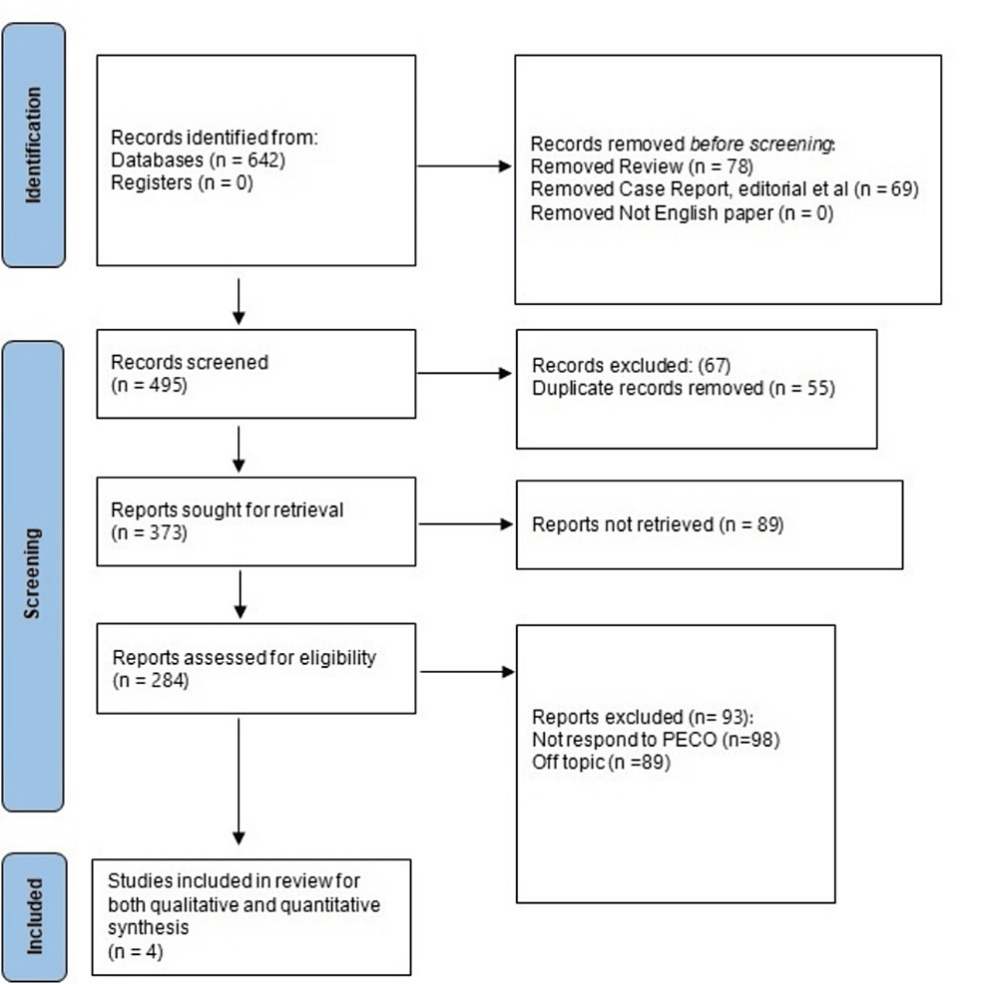
Study selection process for this review

To ensure the study's transparency, thoroughness, and methodological robustness, the Preferred Reporting Items for Systematic Reviews and Meta-Analyses (PRISMA) procedure [15] was meticulously followed, the schematic of which is represented in Figure 1. The systematic search for pertinent literature was made possible in large part by the PRISMA methodology. To ensure the detection of all pertinent studies, these search phrases were methodically merged. Only studies that were pertinent to the study issue were taken into consideration thanks to the clearly stated inclusion and exclusion criteria. The screening procedure was governed by the PRISMA protocol, and papers that met the eligibility requirements were chosen after initial reviews of the titles, abstracts, and full-text evaluations.

The Population, Exposure (or Intervention), Comparison, and Outcome (PECO) Protocol

The PECO protocol for this investigation was designed to frame the research question and guide the study's methodology. It serves as a structured approach to ensure clarity and specificity in research design.

Population (P): The target population for this review included individuals from the Indian population, regardless of age, gender, or other demographic factors, who were diagnosed with or at risk of DS. This population represented the core focus of the study.

Exposure or intervention (E or I): The primary exposure of interest in this review was the presence or colonization of *C. albicans*, a common fungal pathogen, in individuals with DS within the Indian population. This exposure aimed to explore the association between *C. albicans* and DS.

Comparison (C): The comparison group consisted of individuals from the same Indian population who did not have DS or were not colonized by *C. albicans*. This group served as the basis for comparison to evaluate the prevalence and significance of *C. albicans* in individuals with DS.

Outcome (O): The main outcomes assessed in this review were the prevalence and association of *C. albicans* colonization with the presence and severity of DS in the Indian population.

Database Search Protocol

The database search protocol for this systematic review was formulated to ensure a comprehensive search for relevant studies. Eight different databases were utilized to identify pertinent literature. The search strategy employed Boolean operators and Medical Subject Headings (MeSH) keywords to refine and focus the search results effectively as shown in Table 1. The Boolean operators "AND" and "OR" were strategically used to combine search terms to ensure inclusivity while filtering for relevance. MeSH keywords were employed where applicable to enhance search precision. The search strategy was tailored to each database's syntax and structure to optimize the retrieval of relevant studies.

**Table 1:** Utilization of different search strings across different databases

Database	Search String
PubMed	("Stomatitis, Denture"[MeSH] OR "Candida albicans"[MeSH]) AND "India"[MeSH]
Scopus	("Denture Stomatitis" OR "Candida albicans") AND "India"
Embase	("Denture Stomatitis" OR "Candida albicans") AND "Indian Population"
Web of Science	("Denture Stomatitis" OR "Candida albicans") AND "India"
CINAHL	("Stomatitis, Denture"[MeSH] OR "Candida albicans"[MeSH]) AND "Indian Population"
PsycINFO	("Denture Stomatitis" OR "Candida albicans") AND "India"
LILACS	("Stomatitis, Denture"[MeSH] OR "Candida albicans"[MeSH]) AND "Indian Population"
Google Scholar	"Denture Stomatitis" OR "Candida albicans" OR "India"

Inclusion and exclusion criteria

Inclusion Criteria

Population: Studies involving human participants from the Indian population were included. This criterion was essential to focus specifically on the Indian context.

Outcome of interest: Studies reporting on the prevalence, incidence, or association of DS and *C. albicans* infection in the Indian population were considered. This outcome was central to the research question.

Study design: Both observational studies (cross-sectional, case-control, cohort) and clinical trials were eligible for inclusion. These study designs provided diverse data sources for analysis.

Publication status: Published articles in peer-reviewed journals, as well as unpublished grey literature, were included to minimize publication bias.

Language: Studies published in English, as well as relevant studies in other languages with available translations, were considered. This broad language inclusion helped capture a comprehensive range of research.

Exclusion Criteria

Population: Studies conducted outside of India or not specific to the Indian population were excluded. This criterion ensured the alignment of the study with the research focus.

Irrelevant outcomes: Studies that did not report on DS or *C. albicans* infection or their association were excluded.

Animal studies: Animal studies and in vitro experiments were not considered for inclusion, as the focus was on human populations.

Conference abstracts: Conference abstracts, posters, and presentations without full-text articles were excluded due to limited data availability.

Dissertations and theses: Unpublished dissertations and theses were excluded to maintain consistency with the inclusion of peer-reviewed literature.

Evaluation of Bias

**Figure 2:**
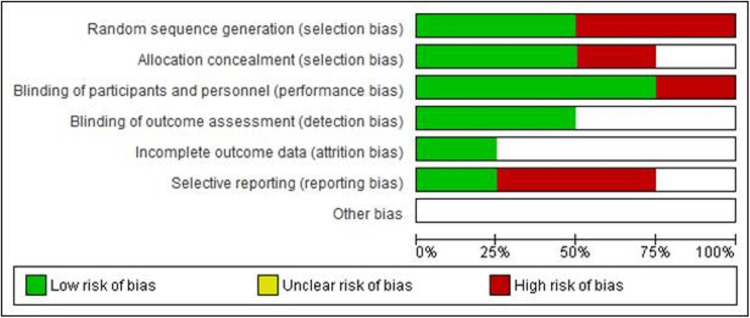
Risk of bias assessment summary across the selected articles

**Figure 3:**
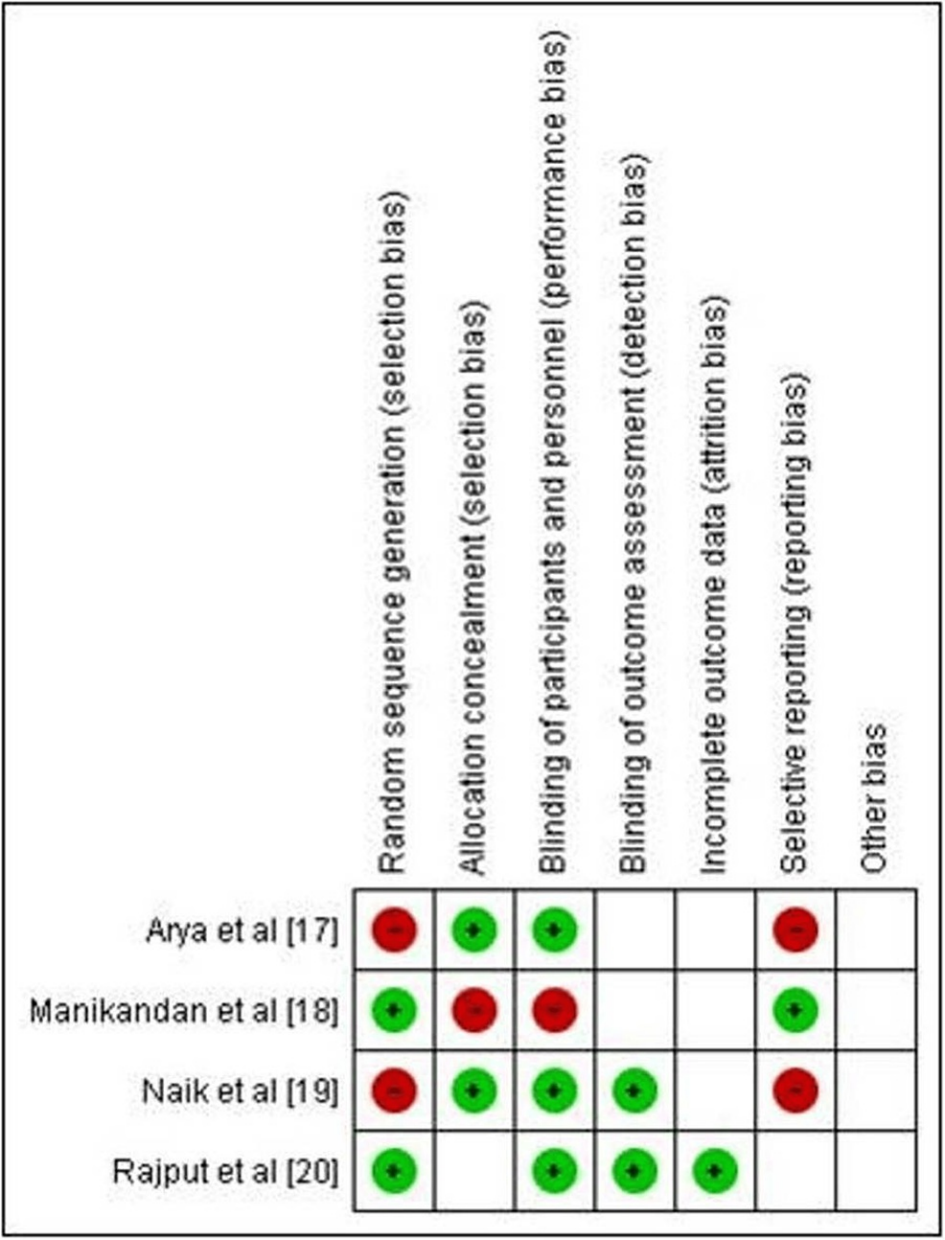
Risk of bias summary graph for the selected papers

To assess the risk of bias in the studies included in this investigation, the Newcastle-Ottawa Scale (NOS) tool [16] was employed. The NOS tool is widely recognized for its utility in evaluating the quality and bias potential of non-randomized studies, including observational studies such as cross-sectional and case-control studies. Figures 2-3 represent the summary and bar graphs for this assessment, respectively.

Meta-Analysis Protocol

The meta-analysis protocol employed for this systematic review was performed using the software RevMan 5 version 5.4.1 (Cochrane, London, United Kingdom).

Results

A total of 642 records were initially identified from various databases. Subsequently, 78 reviews and 69 case reports, editorials, and similar publications were excluded. The inclusion criterion of English-language papers was met by all identified records. Following this initial screening, 495 records remained for further evaluation. Among these 495 records, 55 duplicate entries were removed, ensuring that the analysis was not skewed by redundant data. After duplicate removal, 440 unique records were subjected to a thorough screening process. During the screening, 93 records were excluded for not aligning with the PECO criteria or being off-topic, indicating strict adherence to predefined study criteria. After this comprehensive screening, 284 reports were deemed eligible for inclusion in the review. These reports were subsequently assessed for their suitability for both qualitative and quantitative synthesis. Finally, four studies [17-20] were selected for inclusion in the review, meeting the stringent inclusion criteria established based on the PECO framework.

Table 2 summarizes the demographic variables assessed across the selected studies [17-20].

**Table 2:** Demographic variables assessed across the selected papers

Study ID	Year	Protocol	Sample size (n)	Sex ratio	Mean age (in years)
Arya et al. [17]	2021	Prospective	195	74 females	56.76 ± 12.39
Manikandan et al. [18]	2022	Cross-sectional	60	28 females	68
Naik et al. [19]	2011	Case-control	100	14 females	62
Rajput et al. [20]	2020	Cross-sectional	60	Unspecified	50-70 (range)

These variables include the publication year, study protocol, sample size (n), sex ratio, and mean age (in years) of the participants involved in each study. In terms of the publication years, the studies span from 2011 to 2022. The study protocols employed in the selected papers include prospective, cross-sectional [18,20], and case-control designs [17,19]. The sample sizes in these studies vary, with participant numbers ranging from 60 to 195 individuals. The sex ratio within the studies indicates the gender distribution of participants. Females were more predominant in some studies, while others did not specify the gender distribution. The mean age of participants ranged from 50 to 68 years. This demographic information is crucial as the age of participants can influence the development and severity of denture stomatitis. Older individuals are often more prone to this condition due to factors such as reduced salivary flow and changes in oral mucosa. Table 3 provides a comprehensive overview of the included studies [17-20] that have investigated the presence of *C. albicans* in patients with DS. The table summarizes the aims, parameters assessed, and key inferences observed in these studies.

**Table 3:** Variables assessed pertaining to the presence of C. albicans in patients of DS DS: denture stomatitis, *C. albicans*: *Candida albicans*, *C. krusei*: *Candida krusei*

Study	Aims	Parameters Assessed	Inferences (DS and *C. albicans*)	Other inferences
Arya et al. [17]	Identify the etiology of DS and the role of trauma and fungal infection.	Denture type, presence of stomatitis, presence of *C. albicans*, relationship between stomatitis severity and *C. albicans*.	Significant relation between DS and *C. albicans* in the north Indian population. Slight DS in 52.80% of patients, moderate DS in 32.31%, no DS in 2.56% of subjects. *C. albicans* present in 25.64% of patients.	Unspecified
Manikandan et al. [18]	Compare *C. albicans* isolates in complete denture wearers and nondenture wearers among different age groups.	Denture status, *C. albicans* colony-forming units in saliva culture.	Denture wearers had a higher prevalence of DS associated with heavy growth of *C. albicans* and *C. krusei* in saliva culture. Mean values of *C. albicans *colony-forming units: Group A (*C. albicans* 0.36, *C. krusei* 0.27), Group B (*C. albicans* 0.73, *C. krusei* 0.36).	Unspecified
Naik et al. [19]	Evaluate oral and denture hygiene, salivary measurements, denture age, and *C. albicans* colonization as factors.	Oral and denture hygiene habits, salivary measurements, denture age, degree of *C. albicans* colonization, degree of inflammation.	Denture age correlated with *C. albicans* colonization but not inflammation. Significant differences in *C. albicans* colonization between DS and control groups. *C. albicans* colonization higher in DS patients (53% level 3 contamination) compared to the control group (22% level 3 contamination).	Unspecified
Rajput et al. [20]	Investigate etiological factors in DS in denture-wearing patients.	Denture hygiene, denture stability, denture age, sugar intake, salivation, pH levels, continuous denture wearing.	The overall prevalence of DS was 28% in the study population, with 76% of individuals with DS suffering from *C. albicans* infection.	Factors associated with DS included continuous denture wearing, age of denture, denture hygiene, lack of denture stability, high sugar intake, hypo salivation, and low salivary pH.

**Figure 4:**
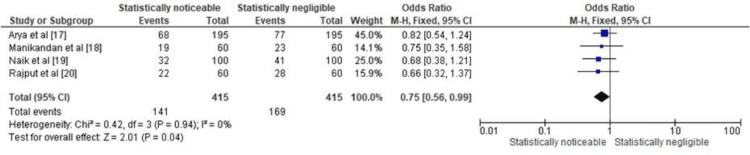
Prevalence of C. albicans in DS patients represented in terms of OR

Figure 4 presents the forest plot depicting the OR for the prevalence of *C. albicans* in patients with DS. The forest plot includes data from all the included papers [17-20], with each study providing information on the number of DS patients with *C. albicans* colonization and the total number of DS patients examined. The forest plot also displays the percentage prevalence of *C. albicans* in each study's DS patient population. The overall result, combining data from all four studies, shows an odds ratio of 0.75 (95% CI: 0.56, 0.99). The 95% CI, which does not cross 1.0, suggests that there is a statistically noticeable association between DS and the presence of *C. albicans*. In other words, DS patients were 25% more likely to have *C. albicans* colonization compared to those without DS. Additionally, the heterogeneity statistics, including the Chi-squared test and I², indicate that there is no significant heterogeneity among the included studies (Chi² = 0.42, df = 3, P = 0.94; I² = 0%). The test for the overall effect, with a Z-value of 2.01 and a P-value of 0.04, further supports the notion that the association between DS and *C. albicans* is statistically significant. Therefore, based on this analysis, it can be concluded that DS is indeed associated with a higher prevalence of *C. albicans* colonization in the studied patient populations.

**Figure 5:**
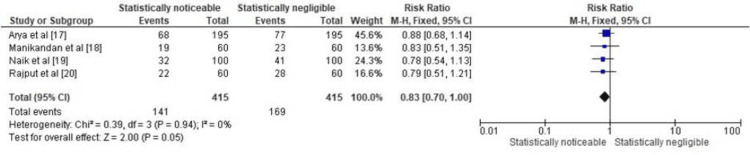
Prevalence of C. albicans in DS patients represented in terms of RR

Figure 5 presents the forest plot displaying the RR for the prevalence of *C. albicans* in patients with DS. The forest plot includes data from all the included papers [17-20], with each study providing information on the number of DS patients with *C. albicans* colonization and the total number of DS patients examined. The forest plot also displays the percentage prevalence of *C. albicans* in each study's DS patient population. The overall result, which combines data from all four studies, yields a relative risk (RR) of 0.83 (95% CI: 0.70, 1.00). The 95% CI, where 1.00 is the lower limit, suggests that there may be a statistically noticeable association between DS and the presence of *C. albicans*. In other words, DS patients may have a slightly higher risk (17%) of *C. albicans* colonization compared to those without DS. The heterogeneity statistics, including the Chi-squared test and I², indicate that there is no significant heterogeneity among the included studies (Chi² = 0.39, df = 3, P = 0.94; I² = 0%). However, the test for the overall effect, with a Z-value of 2.00 and a P-value of 0.05, suggests that while the association between DS and *C. albicans* colonization may not be very strong, it is still on the cusp of statistical significance.

Therefore, based on this analysis, it can be cautiously concluded that DS might be associated with a slightly higher risk of *C. albicans* colonization in the studied patient populations, but further research may be needed to confirm this trend.

Discussion

This review offers crucial information about the prevalence of *C. albicans* colonization and DS in the Indian community. These results highlight the severity of this oral health issue in the area and give crucial information for healthcare professionals, decision-makers, and public health organizations to develop focused interventions and preventive measures. Additionally, the investigation provides insightful data indicating a possible connection between DS and *C. albicans*. Although a relationship is clearly present, the impact size is somewhat small, as seen by the relative risk (RR), which tends to stay close to 1.00. This contradicts earlier beliefs and calls for a more thorough examination to elucidate the relationship. It also implies that DS patients may only have a somewhat decreased risk of *C. albicans* colonization. The review also offers demographic details on DS patients in India, including age distribution, gender prevalence, and mean age. These demographic facts enable a more thorough comprehension of the traits of people at risk for DS, enabling more focused screening and intervention actions.

Moreover, the schematics of the research process conducted for this review exhibit both strengths and areas that could benefit from improvement. One of the notable strengths is the study's strict adherence to the PRISMA guidelines, which ensures transparency and methodological rigor throughout the systematic review. Additionally, the use of a clear and structured PECO protocol provides a well-defined framework for framing the research question and guiding the study's methodology. Furthermore, the study demonstrates a comprehensive approach to database searches by utilizing eight different databases, employing Boolean operators, and incorporating MeSH keywords. This effort reflects a thorough attempt to identify relevant literature. The inclusion and exclusion criteria are also well-defined and aligned with the research focus, contributing to the overall rigor of the study. The utilization of the NOS tool to assess the risk of bias in non-randomized studies is a notable strength, as it enhances the credibility of the review by systematically evaluating study quality and potential bias. Additionally, the use of RevMan 5 software for meta-analysis is a standard and appropriate approach to synthesizing data from the included studies.

Future implications of the findings emphasize the need for more investigation to clarify the relationship between DS and *C. albicans*. Future research should focus on a thorough examination of this relationship, taking into account a variety of elements such as patient demographics, denture kinds, and dental hygiene routines. Establishing causality and improving our understanding of the interactions between DS and *C. albicans* are two things that longitudinal studies can do. For the creation of oral health interventions in India, the knowledge of the incidence of DS and *C. albicans* colonization is also important. Public health initiatives that encourage frequent checkups for denture wearers and encourage good denture hygiene can be crucial in lowering the incidence of DS and related fungal infections. When diagnosing and treating DS patients, dental professionals should also be aware of the possible connection between DS and *C. albicans*. The findings underline the significance of individualized treatment strategies by showing that not all DS cases are inevitably related to *C. albicans*. Moreover, our findings can influence oral health policies in India and direct the distribution of resources for DS care and prevention. Future oral health practitioners will be well-equipped to handle DS and related fungal infections if this information is included in the dental education curriculum.

Arya et al. [17] aimed to explore the etiological aspects of this condition, and they assessed denture type, the presence of stomatitis, the presence of *C. albicans*, and the relationship between the severity of stomatitis and *C. albicans*. Their study found a significant relationship between DS and *C. albicans* in the north Indian population. They also observed varying degrees of stomatitis in their study population, with slight DS in over half of the assessed sample size of patients, moderate DS in 32.31%, and no DS in 2.56% of subjects. Additionally, *C. albicans* was present in 25.64% of patients. Manikandan et al. [18] aimed to compare *C. albicans* isolates in complete denture wearers and nondenture wearers across different age groups. They assessed denture status and the colony-forming units of *C. albicans* in saliva culture. Their findings revealed that denture wearers had a higher prevalence of DS associated with heavy growth of *C. albicans* and *C. krusei* in saliva culture. Moreover, the mean values of *C. albicans* colony-forming units were higher in denture wearers compared to nondenture wearers. Naik et al. [19] conducted an evaluation of oral and denture hygiene, salivary measurements, denture age, and *C. albicans* colonization in DS patients. They assessed oral and denture hygiene habits, salivary measurements, denture age, the degree of *C. albicans* colonization, and the degree of inflammation. Their study found that denture age correlated with *C. albicans* colonization but not with inflammation. Furthermore, they observed a significant difference in *C. albicans* colonization between DS patients and a control group, with a higher level of contamination in DS patients. Rajput et al. [20] investigated the etiological factors contributing to DS in denture-wearing patients. They assessed various factors, including denture hygiene, denture stability, denture age, sugar intake, salivation, pH levels, and continuous denture wearing. Their study reported an overall prevalence of DS of 28% in the study population, with 76% of individuals with DS suffering from *C. albicans* infection. They concluded that factors associated with DS included continuous denture wearing, older denture age, poor denture hygiene, lack of denture stability, high sugar intake, hypo salivation, and low salivary pH.

The included papers also reveal a significant level of geographic and demographic diversity within the country, which has implications for the generalizability of their findings. In the northern part of India, specifically in Uttar Pradesh, Arya et al.'s research [17] identified a substantial relationship between DS and *C. albicans*, emphasizing the role of *Candida* colonization in DS development among the northern Indian population. This suggests that the northern region may experience specific factors contributing to DS. In contrast, Manikandan et al.'s study [18] conducted in southern India (the state of Tamil Nadu) demonstrated regional variations, indicating that denture wearers in the southern part of the country had a higher prevalence of DS linked to *C. albicans*. This geographic diversity highlights potential regional differences in DS etiology.

Moreover, the studies examined different age groups within the Indian population. Manikandan et al. [18] compared age groups of denture wearers, hinting at age-related variations in DS and *Candida* colonization patterns. Additionally, Rajput et al.'s study conducted in Gujarat, the western part of India [20], adds another layer of geographic and demographic diversity to our understanding of DS and *C. albicans* colonization in the Indian population. Gujarat, known for its rich cultural heritage and diverse population, presents a unique context for exploring the etiological factors associated with DS in denture-wearing patients. Therefore, demographic factors like age also contribute to the diversity in findings across these studies. Therefore, despite regional and demographic differences, the generalizability of the findings remains pertinent. The geographic diversity, covering northern, southern, and western India, suggests that while specific findings may be regionally specific, general trends and associations identified, such as the link between DS and *C. albicans*,* *can enrich our broader understanding of DS etiology in the Indian population. These studies collectively provide valuable insights into DS and *C. albicans* colonization within India, allowing for a more comprehensive appreciation of the multifaceted factors contributing to DS across diverse geographic and demographic contexts.

Individuals with compromised immunity, such as those with diabetes or HIV, are at an increased risk of developing DS due to *C. albicans* proliferation. Globally, approximately 65-70% of denture wearers grapple with this issue, with *C. albicans* traditionally recognized as the primary causative species [21-23]. However, recent reports indicate a shifting landscape in this regard, with other fungal species surpassing *C. albicans* in causing these infections. This shift can be attributed to the emergence of drug-resistant strains or mutations within the microbial population, leading to alterations in the microorganism's phenotype. In clinical scenarios, prompt and accurate species-level identification is crucial, as it significantly influences treatment decisions. Numerous authors have underscored *C. albicans* as the predominant species responsible for denture stomatitis [24-27]. Cannon et al. elucidated the capability of this yeast to colonize diverse oral cavity regions, owing to specific interactions with host surfaces, including complement receptors and sugar residues. Microorganisms exhibit a natural propensity for adhering to oral mucosal tissue, leading to potential infections [24-28]. One study [29] expanded on this, indicating that these microorganisms, such as *C. albicans*, not only adhere to the oral mucosa but also establish themselves on inadequately maintained acrylic denture surfaces. The development of biofilms on dentures enhances this adherence, increasing their ability to cause infections [30-36]. It was observed in another article [36] that wearing dentures can exacerbate the adhesion of *C. albicans*.

Limitations

Despite the insightful conclusions drawn from this investigation, there are some limitations that need to be addressed in order to fully comprehend the scope and results of the study. First off, the main obstacle is that there isn't much research on this subject available among Indians. The diversity and representativeness of the data included in the study may have been hampered by the dearth of investigations. As a result, the results may not fully capture the range of DS and *C. albicans* colonization in India, hence, restricting their applicability to other countries. Moreover, the analysis only included a small number of individual studies, which could have weakened the validity of the meta-analysis. The impact size estimations may have been more accurate, and a more complete picture of the association between DS and *C. albicans* may have resulted from the inclusion of a wider and more varied pool of studies. The review also relies heavily on cross-sectional and observational research, which by their very nature limit the ability to establish causal correlations. Although connections were found, definite causality could not be established. To understand the underlying causal mechanisms behind the link between DS and *C. albicans* colonization, prospective longitudinal studies or randomized controlled trials are required.

Highlights of the study

Denture stomatitis is a multifactorial condition seen mostly in the upper jaw, especially on the palatal mucosa as mentioned before. The management of the condition must be comprehensive starting with a proper diagnosis of the causative and risk factors and then directing the treatment toward the most significant factors which are patient-specific.

The investigation revealed important risk factors for DS, such as wearing dentures continuously, having poor denture cleanliness, having unstable dentures, eating a lot of sweets, hyposalivation, and low salivary pH levels besides a commonly believed risk factor, i.e., colonization by *C. albicans*.

The findings underline the significance of individualized treatment strategies by showing that not all DS cases are inevitably related to *C. albicans*. Individuals with compromised immunity, such as those with diabetes or HIV, are at an increased risk of developing DS due to *C. albicans* proliferation

There is a lack of oral health awareness and preventive strategies in the Indian population. There is no efficient health system, especially in dental and oral settings in India.

Estimation of the prevalence of denture stomatitis just on cross-sectional studies doesn’t give any statistically valid results. Hence, one of the main benefits of this research is the precise evaluation spread of denture stomatitis and *C. albicans *by using systematic review and meta-analysis in the country. Furthermore, the findings of the present study can influence oral health policies in India and direct the distribution of resources for DS care and prevention.

## Conclusions

The evaluated results highlight the clinical importance of DS as a prevalent oral ailment among those wearing dentures in India, with an estimated total prevalence of about 28%. The investigation also revealed important risk factors for DS, such as wearing dentures continuously, having poor denture cleanliness, having unstable dentures, eating a lot of sweets, having less saliva, and having low salivary pH levels. These elements work synergistically to influence the onset and course of DS in the Indian population. Additionally, the meta-analysis found statistically significant evidence of a connection between DS and *C. albicans* colonization, which has helped to clarify the role of this yeast's pathogenicity in the development of DS. Despite the fact that the total effect size points to a statistically significant link, it is necessary to consider the potential impact of some restrictions, such as the heterogeneity of the included studies. Additionally, this study emphasizes the significance of oral health awareness and preventive strategies in groups with a high frequency of denture usage in a larger context. Healthcare workers and dentistry professionals in India can learn more about the unique risk factors and prevalence rates connected to DS and *C. albicans* colonization.
